# Mucobilia in Association With a Biliary Cystadenocarcinoma of the Caudate Duct: A Rare Cause of Malignant Biliary Obstruction

**DOI:** 10.1155/2000/48426

**Published:** 2000-01

**Authors:** Ronald S. Chamberlain, Leslie H. Blumgart

**Affiliations:** 1 Hepatobiliary Surgery Service Department of Surgery Memorial Sloan-Kettering Cancer Center New York NY USA; 2 Hepatobiliary Service Department of Surgery 1275 York Avenue New York NY 10021 USA

## Abstract

Mucobilia is a rare condition characterized by the accumulation of abundant mucus within the intra- or extrahepatic biliary tree. A variety of hepatobiliary and pancreatic neoplasms are mucin producing and have been associated with the development of mucobilia including biliary mucinosis, biliary papillomatosis, mucin-producing cholangiocarcinoma (MPCC), or cystic neoplasms of the pancreas or biliary tree (cystadenoma or cystadenocarcinoma). We report the case of 46 year-old male with a biliary cystadenocarcinoma of the caudate lobe which resulted in chronic biliary obstruction and relapsing cholangitis. A review of the literature for both mucobilia and biliary cystadenocarcinoma is provided along with a discussion addressing the clinical presentation, diagnosis, treatment, and prognosis for this rare entity.


*HPB Surgery*, 2000, Vol. 11, pp. 345-351
Reprints available directly from the publisher
Photocopying permitted by license only

© 2000 OPA (Overseas Publishers Association) N.V.
Published by license under
the Harwood Academic Publishers imprint,
part of The Gordon and Breach Publishing Group.
Printed in Malaysia.

### *Case Report*

### Mucobilia in Association with a Biliary Cystadenocarcinoma of the Caudate Duct: A Rare Cause of Malignant Biliary Obstruction

RONALD S. CHAMBERLAIN and LESLIE H. BLUMGART*

*From the Hepatobiliary Surgery Service, Department of Surgery, Memorial Sloan-Kettering Cancer Center, New York, NY*

*(Received 14 July 1999)*

**Mucobilia is a rare condition characterized by the accumulation of abundant mucus within the intra- or extrahepatic biliary tree. A variety of hepatobiliary and pancreatic neoplasms are mucin producing and have been associated with the development of mucobilia including biliary mucinosis, biliary papillomatosis, mucin-producing cholangiocarcinoma (MPCC), or cystic neoplasms of the pancreas or biliary tree (cystadenoma or cystadenocarcinoma). We report the case of 46 year-old male with a biliary cystadenocarcinoma of the caudate lobe which resulted in chronic biliary obstruction and relapsing cholangitis. A review of the literature for both mucobilia and biliary cystadenocarcinoma is provided along with a discussion addressing the clinical presentation, diagnosis, treatment, and prognosis for this rare entity.**

*Keywords:* Biliary cystadenocarcinoma, mucobila, caudate duct

### INTRODUCTION

Mucobilia is a rare condition characterized by the accumulation of abundant mucus within the intra- or extrahepatic biliary tree. The mucus is typically rich in albumin and electrolytes and if treated by external biliary drainage may result in significant electrolyte abnormalities. A variety of hepatobiliary and pancreatic neoplasms are mucin-producing and have been associated with the development of mucobilia [1]. These neoplastic processes include biliary mucinosis, biliary papillomatosis, mucin-producing cholangiocarcinoma (MPCC), or cystic neoplasms of the pancreas or biliary tree (cystadenoma or cystadenocarcinoma) [1-4]. Due to the variety of

*Address for correspondence: Chief, Hepatobiliary Service, Department of Surgery, 1275 York Avenue, New York, NY 10021. Tel.: (212)-639-5536, Fax: (212) 794-5852, e-mail: blumgarl@mskcc.org neoplastic processes that may result in this condition, the clinical spectrum and presentation of patients with mucobilia is varied. This report describes a middle-aged male who developed mucobilia and biliary obstruction due to a biliary cystadenocarcinoma of the caudate duct.

### CASE REPORT

The patient is a 46 year-old white male who was referred for evaluation of a multiloculated cystic lesion arising from the caudate lobe of the liver. For five years, he reported bi-monthly fevers, rigors, and intermittent episodes of jaundice that occurred with increasing frequency in the last three months. Prior ultrasound examinations failed to reveal evidence of gallbladder disease, choledocholithiasis or other liver pathology. The patient was born in the United States with no history of foreign travel. Amoebic and hepatitis serologic tests were negative. Prior to referral, his symptoms had been managed with chronic antibiotic therapy for over two years. Three months earlier, he was hospitalized with jaundice, rigors and fever (40.2 C). Computed tomography (CT) revealed a complex cystic mass in the caudate lobe that was believed to be a pyogenic hepatic abscess. Percutaneous aspiration revealed clear viscous fluid and cytologic analysis showed no malignant cells. Cultures grew both *Enterobacter cloacae* and *Enterococcus faecalis*. After two weeks of antibiotic therapy, his fever persisted and a laparotomy was performed. The cystic lesion was unroofed, and a copious amount of clear mucus abd particulate material was evacuated from the cyst cavity. Histologic review revealed a papillary cystadenoma of biliary origin. Post-operatively, he developed recurrent septic complications, and was referred for further management.

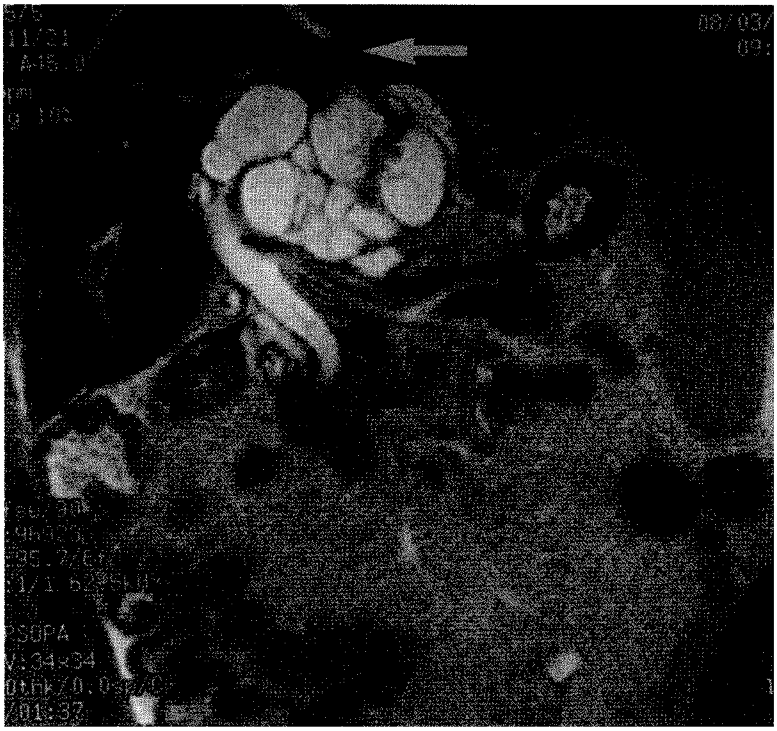

FIGURE 1A Sagittal view from an MRCP sequence demonstrate a complex cystic lesion in communication with the common bile duct (black arrow) arising from caudate lobe inferior to the left lateral segment (Segment III).

At the time of referral, he was jaundiced, pruritic, and febrile (38.7 C). Laboratory evaluation revealed a white blood cell count of 14.7 $cm^3$, a hemoglobin of 9.4 mg/dl, normal serum electrolytes, an AST of 86 mg/dl, an ALT of 121 mg/dl, an alkaline phosphatase of 686 mg/dl and a bilirubin of 8.7 mg/dl. Repeat hepatitis serologies and alpha-fetoprotein (AFP) and carcinoembryonic antigen (CEA) serum levels were normal. On physical examination, the patient was deeply jaundiced with skin changes consistent with chronic urticaria. Bitemporal wasting and loss of skin turgor suggested excessive weight loss and dehydration. The sclera were icteric. Cardiac and pulmonary exams were unremarkable. The abdominal exam revealed a well healed right subcostal incision. No hepatosplenomegaly, masses, or hernias were palpable. A duplex ultrasound (DUS), magnetic resonance cholangiopancreatography (MRCP), and hepatic arteriogram were obtained. The DUS revealed an 8-cm multiloculated cystic mass with internal septations located within the caudate lobe of the liver. Intrahepatic biliary dilatation was present in both the right and left lobes. The MRCP confirmed the presence of a complex cystic caudate lobe mass that appeared to communicate with the caudate duct. (Figs. 1A, B, and C). The arteriogram revealed normal celiac and superior mesenteric arterial anatomy.

At operation, dense adhesions were encountered, and there was early evidence of left lobe atrophy and portal hypertension. A 10 × 12-cm cystic lesion of the caudate lobe extending medially into the base of segment IV, and superiorly into segment II was identified. A left hepatectomy with caudate lobectomy, and cholecystectomy was performed. Following transection of the left hepatic duct, mucus was observed to extrude from the right hepatic duct

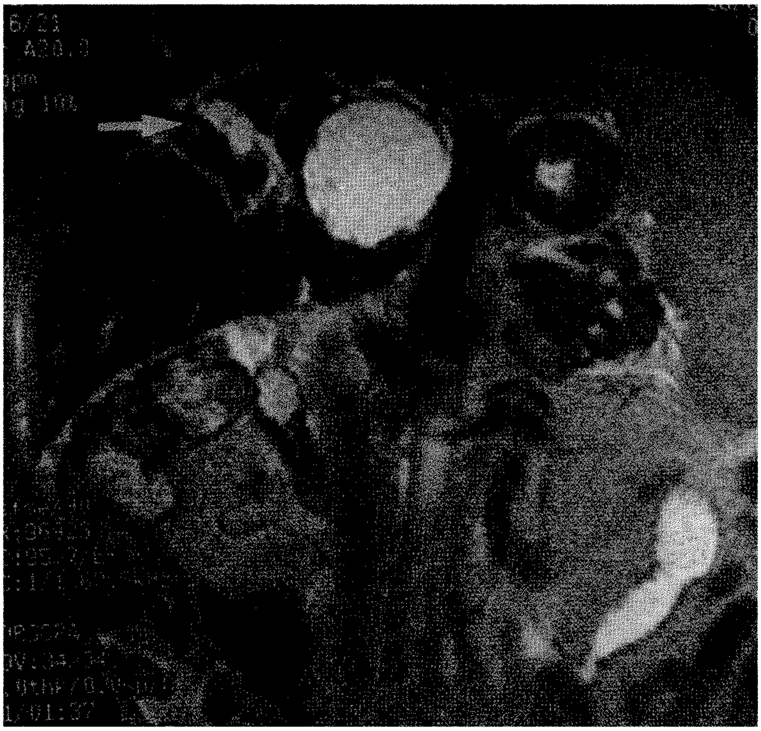

FIGURE 1B Sagittal view from an MRCP sequence demonstrate a large cystic lesion within the caudate lobe (black arrow), displacing the portal structures and causing intrahepatic biliary dilatation (white arrow).

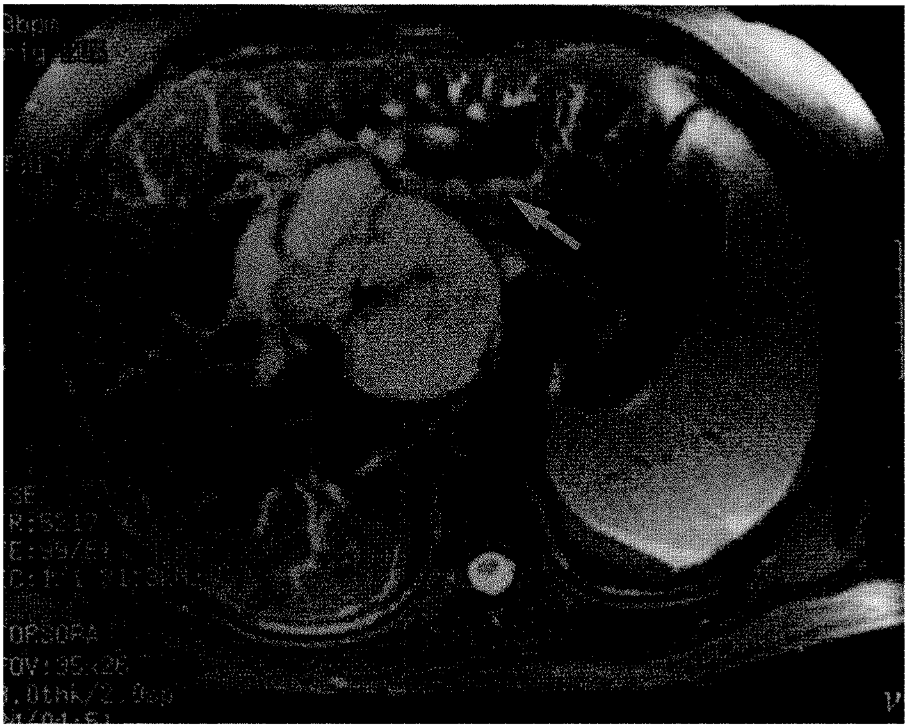

FIGURE 1C T2-weighted MRI image demonstrating the origin of a complex cystic lesion within the caudate lobe. The inferior vena cava (white arrow) is markedly compressed, and the left portal vein displaced to the right (black arrow). Left-sided biliary dilation is very apparent with a prominent and dilated segment III bile duct.

and common bile duct under intense pressure. A pediatric Yankauer suction catheter was used to lavage the biliary tree and remove inspissated mucus in order to restore biliary flow. The patient's post-operative course was prolonged and complicated by a recurrent intra-abdominal biloma and upper gastrointestinal hemorrhage. He was discharged on the 39th post-operative day. At 10 months follow-up he has regained 30 pounds, and returned to an active work and social life. There was no evidence of recurrent tumor.

Pathological review of the specimen revealed a 9 × 14 × 2-cm cystic lesion arising from the caudate duct with a single 6-mm focus of papillary cystadenocarcinoma with oncolytic features. There was massive cystic dilatation of the caudate duct associated with this neoplastic process. No vascular, perineural, liver or extraductal tissue invasion was identified. The resection margins were clear of tumor. The remainder of the liver showed evidence of chronic large duct biliary obstruction. All celiac and periportal lymph nodes were negative for tumor.

### DISCUSSION

Jaundice is a common presentation for many hepatobiliary and pancreatic malignancies and occurs most commonly as a result of compression or blockage of the extrahepatic bile duct by tumor. Jaundice may also occur as a result of direct portal venous invasion and subsequent hepatic atrophy, tumor emboli into the portal vein, intraductal tumor embolus, extrahepatic bile duct metastases, extrinsic compression of the extrahepatic bile duct by nodal metastases, or mucobilia with obstruction of the bile duct by inspissated mucus. Mucobilia has been reported in association with both benign and malignant neoplasms of the pancreas, liver, and bile duct [1–4]. Benign conditions include biliary mucinosis (characterized by mucus metaplasia of the entire extrahepatic biliary tree), biliary papillomatosis (a benign polypoid neoplasm which may involve any part of the biliary tract or gallbladder,) and cystadenomas of either the pancreas or bile duct [3,4]. Of note, even in the setting of biliary mucinosis or papillomatosis in which secretions may be voluminous, biliary obstruction and jaundice are rare events. Malignant conditions that may result in mucobilia include MPCC, and cystadenocarcinomas of the pancreas, liver or biliary tree [5–9]. Twenty cases of mucobilia in association with a MPCC have been reported to date [5–10]. The extent of mucin production in association with MPCC is highly variable, and mucin is generally retained within the tumor cells. Less than 50% of all cases of MPCC associated mucobilia have resulted in jaundice [5–7].

Biliary cystadenomas (BC) and biliary cystadenocarcinomas (BCCA), represent a distinct group of mucin-producing intrahepatic biliary neoplasms that may result in mucobilia and biliary obstruction [2]. Both BC and BCCA are rare cystic tumors constituting less than 5% of all intrahepatic cystic neoplasms [1,11]. Fifty percent of these tumors arise in the right lobe, 29% in the left lobe, and 16% are bilobar [11]. They can arise from either intrahepatic or less commonly extrahepatic bile ducts and occur predominantly in middle-aged women (62%, mean age 56.2 years) [11]. BC and BCCA resemble similar lesions, which may develop within the pancreas and are classified as either multilocular (as in the current case) or papillary, with the former being more common. The clinical presentation is generally mild with right sided abdominal pain or discomfort (57%) being the most common finding followed by an abdominal mass (30%), and elevated liver function tests (26%) [11]. Symptoms such as bone pain, ascites or jaundice are rare and usually indicative of advanced disease [11, 12]. Serologic tumor markers such alphafetoprotein (AFP), carcinoembryonic antigen (CEA), and CA 19–9 are generally not helpful, and are elevated in 8%, 14%, and 36% of patients respectively [11]. Adequate pre-operative assessment of these intrahepatic tumors can be accomplished with a variety of imaging modalities including ultrasound, CT, magnetic resonance imaging (MRI), endoscopic retrograde cholangiopancreatography (ERCP) or percutaneous transhepatic cholangiography (PTC). The sensitivity of these studies to identify BC and BCCA is reported as 100%; the specificity is unknown [11,13]. The typical appearance on either ultrasound or axial imaging (CT or MRI) is a hypodense cystic lesion with internal septa or papillary projections. While not pathognomonic, these findings should suggest the diagnosis of a BC or BCCA. Cholangiocarcinoma more typically present with a solid mass and proximal dilatation, as opposed to BC or BCCA that typically arise from a single duct or multiple ducts with surrounding ectasia and without evidence of proximal obstruction. Diagnosing mucobilia in the absence of endoscopic cholangiography or operative evaluation is difficult, and although some authors have described a characteristic diffuse ground glass appearance to the dilatation of the intrahepatic bile duct, this is not widely accepted [13]. Biliary ductal ectasia limited to one or several adjacent ducts without prominent diffuse ductal dilatation is the characteristic cholangiographic appearance of both BC and BCCA. ERCP or PTC may identify a direct communication between the cyst and individual intrahepatic ducts which can be important in presurgical planning. Hyperechoic spots seen within these dilated ducts, either on ultrasound or axial imaging, may represent mucin but is not diagnostic [13]. Filling defects seen within dilated hepatic ducts may represent a number of conditions including air bubbles, blood clots, tumor nodules from a papillary neoplasm, intrahepatic stones or mucin. CT and ultrasound may be useful in distinguishing between these entities but may not be diagnostic. Precise differentiation between these entities usually requires histopathologic analysis. In the current case, magnetic resonance cholangiopancreatography (MRCP) was utilized to image this lesion in both the axial and sagittal plane. MRCP is capable of not only defining the anatomic location of the tumors within the liver but may also demonstrate a communication between the tumor and the intrahepatic biliary tree without the need for invasive procedures. Fine needle aspiration (FNA), which has been a useful adjunct in the pre-operative evaluation of many hepatobiliary malignancies, is generally contraindicated when a BC or BCCA are suspected. FNA risks not only introducing bacteria into a sterile cyst cavity, but more importantly may result in dissemination of tumor cells.

Ishak *et al.*, reported fourteen cases of BCCA and identified a single case of mucobilia due to either a BC or BCCA [14]. Lauffer *et al.*, reviewed 113 cases of BCCA reported in the literature up until 1998 and reported on sixteen patients presenting with jaundice [11]. A precise cause for the jaundice in these cases was not provided, however the authors did state that it was due to either tumor compression of the extrahepatic bile duct, parenchymal displacement by tumor, or rarely mucus hypersecretion. Kokubo *et al.*, reported on six patients with mucobilia resulting from a mucin-hypersecreting intrahepatic biliary neoplasm [7]. Four of the six cases were BCCA, one was a MPCC, and in one case the pathology was indeterminate. In three cases of BCCA, jaundice was the presenting complaint.

Macroscopically, BCCA are typically multiloculated (84%) cysts lined by papillary adenocarcinoma [1, 11]. Histologically, BCCA differ from BC, in that they possess a cellular pleomorphism and anaplasia with infiltration of the underlying fibrostroma. The malignant epithelium is typically multi-layered with numerous papillary projections, with breaks or erosion in the basement membrane. Benign columnar epithelium is observed in nearly all cases (91%), and was present in the current case [1, 11]. The presence of benign cystic epithelium adjacent to malignant epithelium supports most pathologists' belief that BCCA arise within BC. BCCA are generally large (mean 12.4 cm), with a reported range of 1.2 to 30 cm [11]. Invasion into blood vessels, adjacent liver or nearby organs occurs in roughly half of all cases (52%), with distant metastases (20%), and lymphatic spread (13%) occurring infrequently [1, 11]. Wolf *et al.*, has recently described an oncocytic and non-oncocytic variant of BCCA that may have prognostic implications [15]. Oncocytic epithelial cells, or oncocytes, are cells with abundant, bright eosinophilic and granular cytoplasm and exuberant numbers of mitochondria. These cells have been described in malignancies of the pancreas, salivary glands, ovary, and breast, and in these cases the non-oncocytic tumor population determines the potential for tumor progression. In the current case, the invasive component of the tumor was 6-mm in size, and was noted to resemble those of an intraductal oncocytic papillary carcinoma of the pancreas that is usually associated with a favorable prognosis [16].

Similar to other causes of malignant biliary obstruction, surgical resection is standard therapy for BCCA (with or without mucobilia). Patients otherwise fit for operation and without evidence of lymph node or distant metastases should be explored. It is our practice to perform a diagnostic laparoscopy on all patients with a suspected biliary malignancy to exclude the presence of obvious extrahepatic disease. If the location of the offending lesion has not been clearly delineated pre-operatively, significant effort should be aimed at identifying the location of the lesion to ensure it is included within the resected specimen. Excessive mucorrhea may limit the ability of intraoperative ultrasound or bi-manual palpation of the liver to provide helpful information in identifying tumor location. In these instances, choledochoscopy may be useful in not only providing direct tumor visualization, but also allowing for biopsy and histologic diagnosis. However, given the difficulty in differentiating between reactive atypia and a malignant neoplasm on frozen section, and the potential for malignant transformation of many biliary neoplasms, a benign intraoperative histologic interpretation should not discourage an attempt at complete surgical resection with negative margins. Since mucus may also result in obstruction of the bile duct on the contralateral side of the offending lesion, intraoperative lavage of the entire biliary tree to remove offending mucus plugs is an important part of the procedure. Free flow of bile from the contralateral lobe of the liver should be restored and is vitally important when pre-operative biliary intubation has occurred, since bactobilia in these patients approaches 100%. The overall 5-year survival for patients with a BCCA undergoing surgical resection is 65–71%. If a complete surgical resection with negative histologic margins is achieved, a two- and five-year survival rate of 100%, and a recurrence rate of 13% has been reported [1,11].

### CONCLUSIONS

Mucobilia is a rare cause of malignant biliary obstruction associated with a number of benign and malignant mucin-producing pancreatic and biliary neoplasms. Although radiologic imaging of the biliary tree cannot provide a definitive diagnosis of obstruction due to mucin, intrahepatic biliary ductal dilatation in association with jaundice, with or without the presence of a solid tumor within the biliary tree, should prompt one to consider this diagnosis. The presence or absence of mucobilia does not change the algorithm for dealing with patients with malignant biliary tract obstruction due to any cause; surgery remains the mainstay of therapy. Complete resection with negative histologic margins may be curative in nearly all cases of BCCA.

### *References*

[1] Chen, M.-F. (1998). Mucobilia: The clinical spectrum, diagnosis and treatment. *J. Gastroenterol Hepatol.*, **13**, 1084–90.

[2] Tsiftis, D., Christodoulakis, M., de Bree, E. and Sanidas, E. (1997). Primary intrahepatic biliary cystadenomatous tumors. *J. Surg. Oncol.*, **64**, 341–6.

[3] Caroli, J., Soupault, R. and Champaeu, M. (1959). Papillomes et papillomatoses de la voie billiaire principale. *Rev. Med. Chir. Mal. Foie.*, **34**, 191–5.

[4] Goinard, P., Pelissier, G. and Streit, P. (1960). Mucinose des voies biliaries. *Mem. Acad. Chir.*, **86**, 849–54.

[5] Hadjis, N. S., Slater, R. N. S. and Blumgart, L. H. (1987). Mucobilia: An unusual cause of jaundice. *Br. J. Surg.*, **74**, 48–9.

[6] Styne, P., Warren, G. H., Kumpe, D. A. *et al.* (1986). Obstructive cholangitis secondary to mucous secreted by a solitary papillary bile duct tumor. *Gastroenterology*, **90**, 748–53.

[7] Kokubo, T., Itai, Y., Ohtomo, K. *et al.* (1988). Mucinous-hypersecreting intrahepatic biliary neoplasms. *Radiology*, **168**, 609–14.

[8] Jan, Y. Y., Chen, M. F. and Hung, C. F. (1977). Cholangiography of mucin-hypersecreting intrahepatic biliary neoplasms. *J. Hep. Bil. Pancr. Surg.*, **4**, 180–4.

[9] Jan, Y. Y., Chen, M. F. and Chen, T. J. (1994). Cholangiocarcinoma with mucobilia. *J. Formous. Med. Assoc.*, **93**, S149–55.

[10] Capizzi, P. J., Rosen, C. B. and Nagorney, D. M. (1992). Intermittent jaundice by tumor emboli from intrahepatic cholangiocarcinoma. *Gastroenterology*, **103**, 1669–73.

[11] Lauffer, J. M., Baer, H. U., Maurer, C. A. *et al.* (1998). Biliary cystadenoma of the liver: the need for complete resection. *Eur. J. Cancer.*, **34**, 1845–51.

[12] Lemoto, Y., Kondo, Y. and Fukamachi, S. (1981). Biliary cystadenocarcinoma with peritoneal carcinomatosis. *Cancer*, **48**, 1664–7.

[13] Takayasu, K., Muramatsu, Y., Moriyama, N. *et al.* (1988). Imaging diagnosis of the bile duct of cystadenocarcinoma. *Cancer*, **61**, 941–6.

[14] Ishak, K. G., Willis, G. W., Cummins, S. D. and Bullock, A. A. (1977). Biliary cystadenoma and cystadenocarcinoma: Report of 14 cases and review of the literature. *Cancer*, **39**, 322–38.

[15] Wolf, H. K., Garcia, J. A. and Bossen, E. H. (1992). Oncocytic differentiation in intrahepatic biliary cystadenocarcinoma. *Mod. Path.*, **5**, 665–8.

[16] Adsay, N. V., Adair, C. F., Heffess, C. S. *et al.* (1996). Intraductal oncocytic papillary neoplasms of the pancreas. *Am. J. Surg. Pathol.*, **20**, 980–4.

